# Anterior Mediastinal Lymph Node Metastasis of Intrahepatic Cholangiocarcinoma: A Case Report and Literature Review

**DOI:** 10.70352/scrj.cr.24-0025

**Published:** 2025-01-31

**Authors:** Tomoaki Tabata, Ryusuke Saito, Takeki Taniguchi, Kyohei Kasuda, Naruhito Takido, Hiroyuki Ogasawara, Yoshihiro Shono, Muneyuki Matsumura, Kengo Sasaki, Atsushi Fujio, Kazuaki Tokodai, Takanori Morikawa, Michiaki Unno, Takashi Kamei

**Affiliations:** 1 Department of Surgery, Tohoku University Graduate School of Medicine, Sendai, Miyagi, Japan; 2 Department of Clinical Medicine, Sendai City Hospital, Sendai, Miyagi, Japan; 3 Department of Gastroenterological Surgery, Miyagi Cancer Center, Sendai, Miyagi, Japan

**Keywords:** intrahepatic cholangiocarcinoma, anterior mediastinal metastasis, CA19-9, chemotherapy

## Abstract

**INTRODUCTION:**

Intrahepatic cholangiocarcinoma (ICC) is the second most common liver malignant tumor with a poor prognosis. Lymph node (LN) metastasis is found in 15% of ICC at the time of initial diagnosis. However, the LN metastasis to the anterior mediastinum is extremely rare. Herein, we report a case of anterior mediastinal LN metastasis of ICC.

**CASE PRESENTATION:**

The patient is a 74-year-old man who had surgery for cervical esophageal cancer. During follow-up, a low-density hepatic tumor and swollen LNs in the anterior mediastinum were detected. The tumor of the liver was diagnosed as ICC by needle biopsy. Excisional biopsy of the LN was performed and the diagnosis was metastasis of ICC. Because the prognosis of the patient with ICC Stage IVB is poor, the patient received 8 courses of chemotherapy. Although the new lesion appeared next to the main tumor, these tumors were located in the left liver. In addition, it was difficult for the patient to continue the chemotherapy due to the renal dysfunction. Hepatectomy with lymphadenectomy was performed. The patient survives without recurrence for 9 months after surgery. This is the first report of anterior mediastinal metastasis of ICC without any other organ involvement.

**CONCLUSIONS:**

Metastasis to the anterior mediastinum of hepatic tumor can be explained by the system that lymphatic fluid running under the capsule of the liver drains to the anterior mediastinal LNs through the coronary ligament. Metastasis of ICC to mediastinal LNs can occur when the tumor is located at the surface of the liver. Excisional biopsy is effective in determining the accurate disease stage and the treatment strategy.

## Abbreviations

CA19-9
: carbohydrate antigen 19-9
CEA
: carcinoembryonic antigen
CT
: computed tomography
EOB-MRI
: ethoxybenzyl-magnetic resonance imaging
FDG-PET
: fluorodeoxyglucose–positron emission tomography
HCC
: hepatocellular carcinoma
ICC
: intrahepatic cholangiocarcinoma
LN
: lymph node
SCC
: squamous cell cancer
SUV
: standardized uptake value

## INTRODUCTION

Intrahepatic cholangiocarcinoma (ICC) is the second most common liver malignant tumor with a poor prognosis.^1)^ It is reported that ICC accounts for 20% of hepatic malignancies and its incidence has been increasing recently.^2)^ Although there are some advanced treatment options including chemotherapy and radiotherapy, surgery is still the primary method to cure ICC, and the 5-year survival rate of ICC is only 30%.^3)^

Lymph node (LN) metastasis is found in 15% of ICC at the time of initial diagnosis.^4)^ Preoperative imaging study using computed tomography (CT) and fluorodeoxyglucose–positron emission tomography (FDG-PET) is important because LN metastasis would influence the treatment strategy and prognosis.^5)^ However, the therapeutic benefit of lymphadenectomy is still controversial.^6)^

Anterior mediastinal LN metastasis is often observed in thyroid and lung cancer.^7,8)^ Metastasis from other origins is extremely rare. In this report, we confirmed the anterior mediastinal metastasis of ICC by excisional biopsy at the initial diagnosis. In addition, the patient survives without recurrence for 9 months after surgery with the combined modality therapy.

## CASE PRESENTATION

**Fig. 1:**
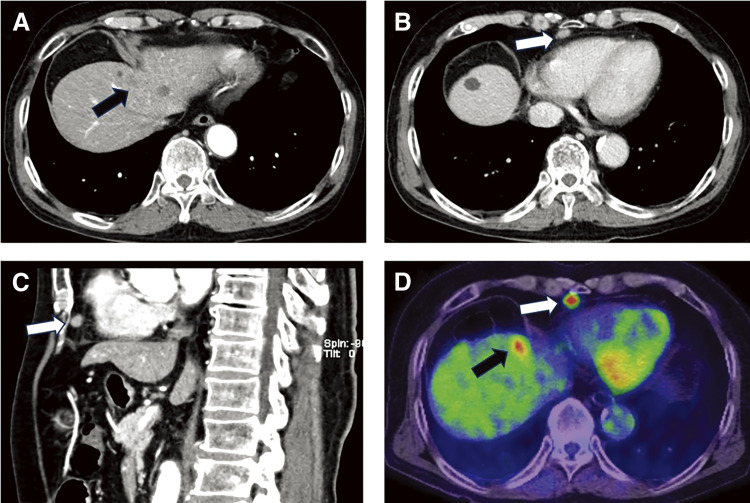
Contrast-enhanced CT and FDG-PET. (**A**) CT showed a 15-mm-large, low-density lesion in S4 of the liver. (**B**) CT demonstrated the enlarged lymph node in the anterior mediastinum (axial view). (**C**) Anterior mediastinal lymph node (sagittal view). (**D**) The accumulation of FDG in the lymph node and the hepatic lesion was observed by PET. Black arrow: hepatic lesion; white arrow: metastatic lymph node

**Fig. 2:**
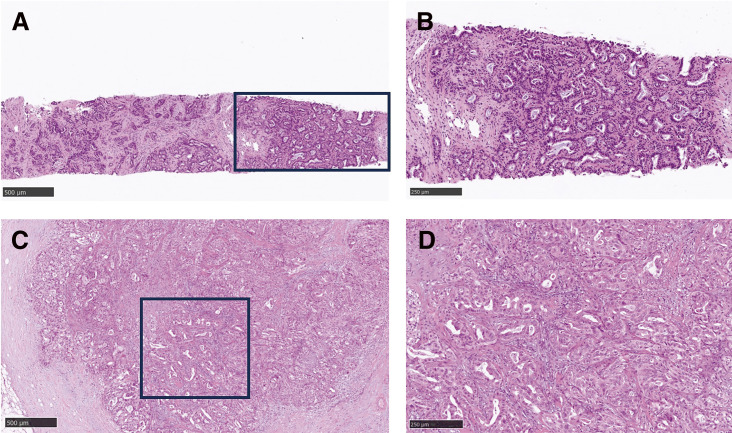
HE staining of the hepatic lesion and the lymph node. (**A**) HE staining of the hepatic tumor (low magnification, scale bar: 500 μm). (**B**) HE staining of the hepatic tumor (high magnification, scale bar: 250 μm). (**C**) HE staining of the lymph node (low magnification, scale bar: 500 μm). (**D**) HE staining of the lymph node (high magnification, scale bar: 250 μm). HE, hematoxylin and eosin

**Fig. 3:**
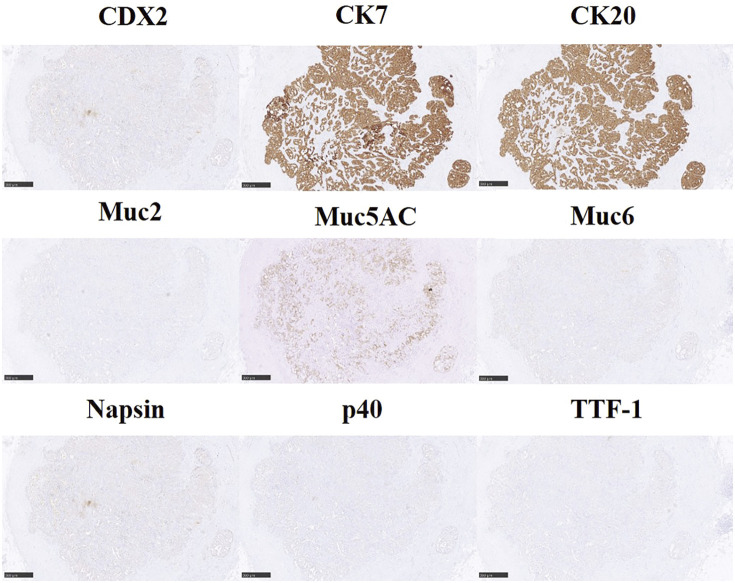
Immunohistochemistry of the lymph node. The tumor of the lymph node was positive for CK7 and CK20 and weakly positive for Muc5AC. Scale bars: 500 μm.

**Fig. 4:**
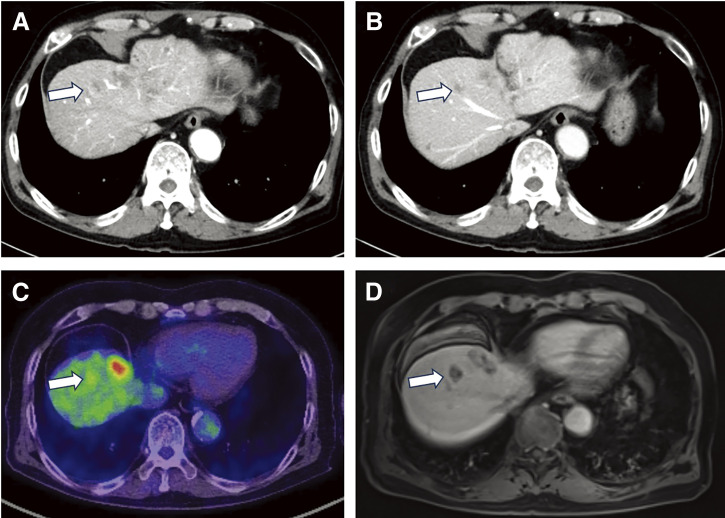
Preoperative images by CT, PET, and MRI. (**A**) CT (portal phase). (**B**) CT (equilibrium phase). (**C**) PET. (**D**) Ethoxybenzyl–magnetic resonance imaging (EOB-MRI). white arrow: new lesion.

**Fig. 5:**
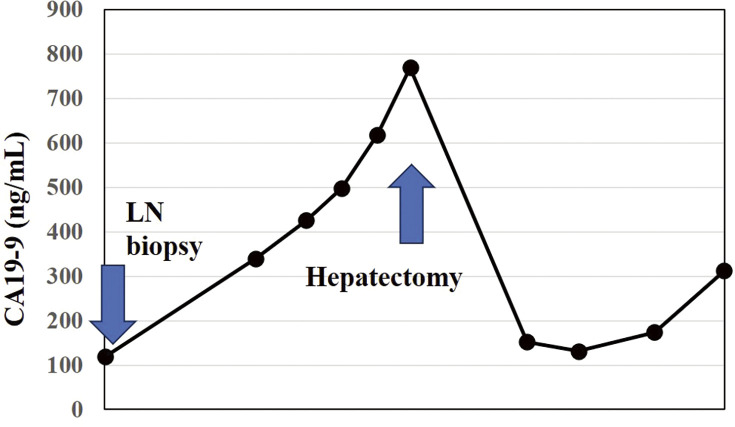
Transition of CA19-9.

**Fig. 6:**
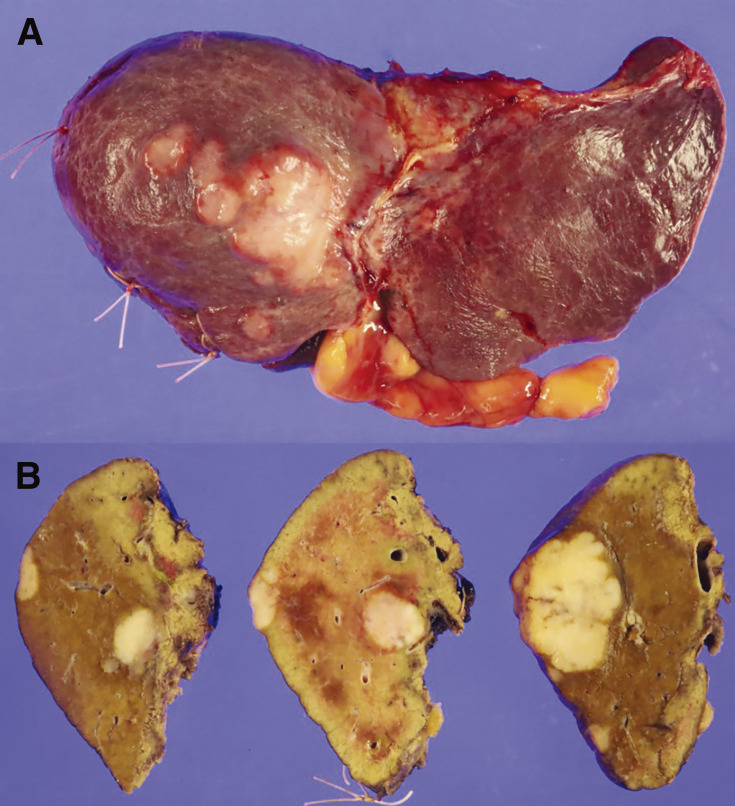
Pictures of the left lobe of the liver. (**A**) Resected specimen. (**B**) Cross-sections of the specimen.

The patient is a 74-year-old man who had surgery for cervical esophageal cancer 2 years ago. The final diagnosis was moderately differentiated squamous cell carcinoma (SCC) with LN metastasis, pT3N1M0 pStage III. Chemoradiotherapy was performed and there was no recurrence of the esophageal cancer. During follow-up, a low-density lesion, a diameter of 15 mm, was detected in segment 4 of the liver by CT (**Fig. 1A**). The tumor was located just adjacent to the umbilical portion. There were some swollen LNs in the anterior mediastinum (**Figs. 1B** and **1C**). The accumulation of FDG was also detected in the hepatic lesion (standardized uptake value; SUV_max_: 8.4) and the anterior mediastinal LN (SUV_max_: 11.1) by PET (**Fig. 1D**). Needle biopsy was performed for the liver tumor and the diagnosis was ICC (**Figs. 2A** and **2B**). The elevation of the serum level of carbohydrate antigen 19-9 (CA19-9) was detected and its level was 119 ng/mL. The levels of carcinoembryonic antigen (CEA) and SCC were within normal range. To confirm the diagnosis of the swollen LNs in the anterior mediastinum, an excisional biopsy was performed. In the operation, there were several firm LNs pulpable just behind the sternum. According to the pathological findings, the tumor in the LN was an intermediate differentiated adenocarcinoma. Atypical cells with increased nuclear chromatin and enlarged nuclei were proliferating and forming glandular structures (**Figs. 2C** and **2D**). The structure of the tumor in the LN was similar to that of the hepatic lesion. Immunohistochemistry showed that the tumor was positive for CK7 and CK20, weakly positive for MUC5AC, but negative for CDX-2, Napsin, TTF-1, and p40 (**Fig. 3**). The final diagnosis was the lymphatic metastasis from ICC. As the diagnosis of distant metastasis (Stage IVB), the chemotherapy using Gemcitabine, Cisplatin, and Durvalumab was performed for 8 courses. The new lesion appeared just next to the main tumor in S4 (**Fig. 4**) and the elevation of CA19-9 was also observed (**Fig. 5**). We could not detect any swollen LNs in the anterior mediastinum by PET or CT. In addition, it was difficult to continue the chemotherapy due to the renal dysfunction. As the tumors were located in the left lobe of the liver, a left hepatectomy with LN dissection was performed. In the operation, there were multiple intrahepatic metastatic lesions limited to the left lobe of the liver. Grossly, the lesion corresponds to a mass-forming type with a central necrotic area and several satellite nodules (**Fig. 6**). The size of the main tumor was 30 mm in diameter. Most of the tumor cells were viable and they showed serosal and neural invasion around the portal vein (S1, Vp1, Vv0, and Va0). There were no metastases in the regional LNs. Although re-elevation of CA19-9 is observed after hepatectomy (**Fig. 5**), the patient does not have any recurrence for 9 months after hepatectomy.

## DISCUSSION

LN metastasis of ICC is one of the greatest contributors to the negative clinical impact with a worse prognosis regardless of the LN dissection.^9)^ Most hepatic lymph flows into the regional LNs in the hepatic hilum through the portal triads.^10)^ The metastasis of ICC to the mediastinal LNs is extremely rare. To our knowledge, this is the first report of anterior mediastinal metastasis of ICC without any other organ involvement. We searched the articles with the keywords “mediastinal metastasis” and “cholangiocarcinoma” using PubMed and summarized the articles in **Table 1**.^11–14)^ There were 4 articles and 1 was a simultaneous solitary LN metastasis from mixed hepatocellular carcinoma (HCC) at the initial diagnosis and the other 3 were recurrences of ICC. There were several articles that HCC metastasized to the mediastinal LN and surgical intervention could be beneficial for these populations.^15)^ Interestingly, although there were no metastases in the regional LNs at hepatectomy, the tumor invaded the round ligament. Although the direct or lymphatic invasion toward the anterior mediastinum could not be confirmed by the pathological findings, it was suspected that micro lymphatic invasion caused the metastasis to the anterior mediastinal LNs. In our and Yoshida’s cases,^11)^ the tumor might have spread directly to the anterior mediastinum through the coronary ligament and diaphragm. In these 2 cases, the tumors were located at the surface and near the diaphragm.

**Table 1:** Previous reports of the intrahepatic cholangiocarcinoma with mediastinal lymph node metastasis

Citation	Sex	Age	Location (liver)	Diameter (mm)	CA19-9 (ng/mL)	Location (mediastinum)	Other metastases	Follow-up period (months)	Recurrence	Mortality
Our case	Male	74	S4	15	119	Anterior	None	6	None	Alive
Yoshida et al.^11)^	Female	77	S8	20	N/A	Anterior	None	6	None	Alive
Kanemitsu et al.^12)^	Female	77	S8	22	2023	N/A	Regional LN	20	Mediastinal LN	Deceased
Komatsu etal.^13)^	Female	59	Left lobe	80	4088	Anterior	Regional LN	64	Paraaortic Mediastinal LN	Alive
Biroulet et al.^14)^	Male	46	S7/8	70	Normal	Posterior	None	36	Bone Mediastinal LN	Deceased

N/A, not addressed; LN, lymph node

It is reported that there are 3 lymphatic vascular systems in the liver.^16)^ The first is the portal tract that drains the lymph to the LNs at the hepatic hilum. The second is the lymphatic vessels that run with the hepatic vein, along the inferior vena cava through the diaphragm toward posterior mediastinal LNs. The last one is the lymphatic fluid running under the capsule of the liver drains to the anterior mediastinal LNs through the coronary ligament. In the case of the tumor located at the surface, the third route which is called the superficial system should be considered.

The most common origin of the mediastinal LN metastasis is lung cancer.^8)^ The origin of the abdominal organs accounts only for 0.8% to 2.3%.^17)^ In this article, urinary tract cancer accounts for 72%, followed by colorectal and ovary cancer. Most of the metastases were observed in the posterior mediastinum, which was thought to occur along with the paraaortic LN metastasis. Therefore, the anterior mediastinal LN metastasis from the abdominal organs is extremely rare.

It is difficult to diagnose the LN metastasis accurately before surgery. The sensitivity and specificity of PET for the diagnosis of metastatic LNs for ICC are reported to be 31.6%–70% and 88.2%–91.7%, respectively.^18,19)^ In our case, only 1 LN was detected as positive for PET preoperatively, but all the resected LNs were positive for metastasis by the pathological findings (N: 4/4). Therefore, we assumed that there could be other metastatic lesions that were not detected by PET. Stage IV ICC patients have very poor survival outcomes and the median overall survival is only 11.7 months with chemotherapy.^20)^ After 6 months of chemotherapy, the patient had hepatectomy because the lesions detected by CT, PET, and MRI were limited to the left lobe of the liver. In addition, there were no swollen LNs in the anterior mediastinum. Multidisciplinary treatment including surgery and chemotherapy is important for a better prognosis.

## CONCLUSIONS

We experienced the ICC with the anterior mediastinal metastasis treated with chemotherapy and surgery. We should keep in mind that the metastasis of ICC to mediastinal LNs can occur when the tumor is located at the surface of the liver. Excisional biopsy is effective in determining the accurate disease stage and the treatment strategy.

## DECLARATIONS

### Funding

No funding was received for this report.

### Authors’ contributions

T Tabata, RS, T Taniguchi, and KK participated in all aspects of this study, including patient management, report conceptualization, and draft writing.

NT, HO, YS, MM, KS, and AF managed the patients.

KT, KM, TM, and TK supervised the article.

All authors have read and approved the manuscript and agree to be held accountable for all aspects of this report.

### Availability of data and materials

The datasets supporting the conclusions of this study are included in this article.

### Ethics approval and consent to participate

Informed consent to participate in this study was obtained from the patient.

### Consent for publication

Consent was obtained from the patient for the publication of this case report.

### Competing interests

All authors declare no competing interests in this article.
